# Effectiveness of Ivabradine in Treating Stable Angina Pectoris

**DOI:** 10.1097/MD.0000000000003245

**Published:** 2016-04-08

**Authors:** Liwen Ye, Dazhi Ke, Qingwei Chen, Guiqiong Li, Wei Deng, Zhiqin Wu

**Affiliations:** From the Department of Geriatrics Cardiology, The Second Affiliated Hospital of Chongqing Medical University, Chongqing, China.

## Abstract

Many studies show that ivabradine is effective for stable angina.

This meta-analysis was performed to determine the effect of treatment duration and control group type on ivabradine efficacy in stable angina pectoris.

Relevant articles in the English language in the PUBMED and EMBASE databases and related websites were identified by using the search terms “ivabradine,” “angina,” “randomized controlled trials,” and “Iva.” The final search date was November 2, 2015.

Articles were included if they were published randomized controlled trials that related to ivabradine treatment of stable angina pectoris.

Patients with stable angina pectoris were included.

The patients were classified according to treatment duration (<3 vs ≥3 months) or type of control group (placebo vs beta-receptor blocker). Angina outcomes were heart rate at rest or peak, exercise duration, and time to angina onset.

Seven articles were selected. There were 3747 patients: 2100 and 1647 were in the ivabradine and control groups, respectively. The ivabradine group had significantly longer exercise duration when they had been treated for at least 3 months, but not when treatment time was less than 3 months. Ivabradine significantly improved time to angina onset regardless of treatment duration. Control group type did not influence the effect of exercise duration (significant) or time to angina onset (significant).

Compared with beta-blocker and placebo, ivabradine improved exercise duration and time to onset of angina in patients with stable angina. However, its ability to improve exercise duration only became significant after at least 3 months of treatment.

## INTRODUCTION

Coronary heart disease (CHD) is a common and often dangerous condition that is typically initially manifested by angina. Treatments for angina that alleviate its symptoms are needed because it severely limits patient quality of life. An insufficient supply of oxygen forms the pathological–physiological basis of angina pectoris, and the heart rate is one of the determinants of myocardial oxygen consumption. Thus, one treatment strategy for angina pectoris is to slow the heart rate. Some existing drugs that slow heart rate such as beta-blockers (BBs) and nondihydropyridine calcium channel blockers also have adverse effects that prevent their use in the treatment of patients with some other diseases. In recent years, the electrophysiology of sinoatrial node activities has become better understood, which has prompted the development of new drugs to control heart rate. One of these drugs may be ivabradine, whose electrophysiological mode of action has become an area of intense research recently. Ivabradine acts on sinus node cells and specifically inhibits the pacemaker I_f_ current, thereby slowing the rhythm of the sinus node and therefore the heart rate. Thus, unlike BB, ivabradine slows the heart rate without inhibiting inner heart conduction^1^ or reducing the left ventricular systolic function.^2^

Many studies have demonstrated that ivabradine effectively treats stable angina.^3–9^ However, since many of these studies assessed clinical ivabradine effectiveness for a short time only, it remains unclear how effective this drug is over longer periods of treatment. The effect of different types of control groups (placebo and BB) on ivabradine efficacy in stable angina also remains unclear. To address these questions, the present meta-analysis of randomized controlled trials (RCTs) on ivabradine treatment for stable angina pectoris was performed.

## METHODS

### Data Sources and Search Strategy

Relevant articles in the English language in the PUBMED and EMBASE databases and related websites were identified by using the search terms “ivabradine,” “angina,” “randomized controlled trials,” and “Iva.” There were no limitations on publication date or publication status. The final search date was November 2, 2015.

### Inclusion and Exclusion Criteria

To select the appropriate articles, the following inclusion and exclusion criteria were used. Articles were included if they were published RCTs that related to ivabradine treatment of stable angina pectoris; articles were in English language; the experimental group was treated with ivabradine for stable angina pectoris and the control group was managed by a routine treatment strategy without ivabradine; the curative effect of ivabradine was assessed by measuring the following 4 endpoints: heart rate at rest, heart rate at peak, exercise duration, and time of onset of angina; and the 4 endpoints were expressed as mean ± standard deviation (SD). Studies were excluded if they were not randomized trials or lacked a control group, or if the full text of the article could not be retrieved.

### Data Extraction and Management

The data of basic information on the patients (Table 1) and the endpoints used in the meta-analysis were extracted by 2 researchers (LY and DK). All RCTs that met the inclusion criteria were included in the meta-analysis. Due to the limitations of the RCTs, some RCTs involved several ivabradine groups, whereas there was only 1 control group.^4,7,9^ As a result, the control group data had to be calculated repeatedly when analyzing the data. To make full use of the data, we treated 12 weeks as 3 months.^6^ To assess the effectiveness of ivabradine in stable angina pectoris, the patients were grouped according to whether they were followed up for less than or at least 3 months. In a separate analysis, the patients were grouped according to whether the control group received a placebo or BB.

**TABLE 1:**
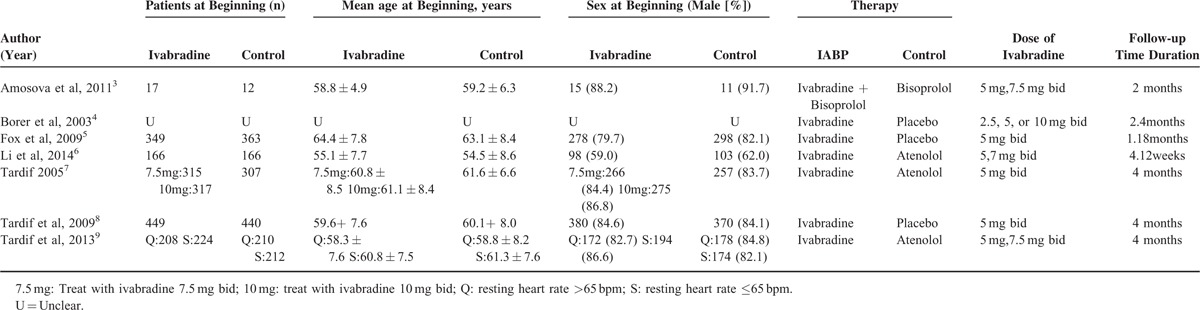
Characteristics of the Included Articles

### Methodology/Quality Assessment

**FIGURE 1:**
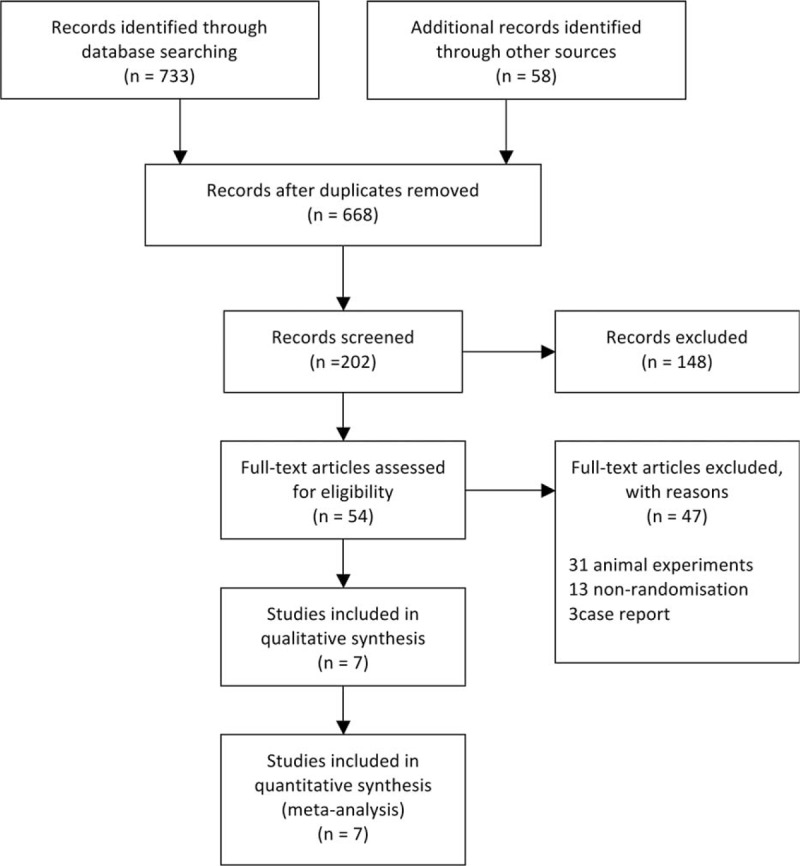
The numbers of studies that were screened, assessed for eligibility, and included in the meta-analysis. The reasons for excluding certain studies at each stage are also shown.

The numbers of studies that were screened, assessed for eligibility, and included in the meta-analysis are shown in the flow diagram in Figure 1, along with the reasons why certain studies were excluded at each stage, as recommended by the PRISMA Statement.^10^ Furthermore, a 27-item checklist (Supplemental Checklist) for transparent reporting of a systematic review were used to be included in the study. The 7 selected RCTs were assessed by 2 reviewers according to the Cochrane Collaboration bias risk tool. Six aspects were mainly involved: random sequence generation, allocation concealment, double blinding, incomplete outcome data, selective reporting, and other biases. All these trails were RCTs obtained by the single-blind method^3^ or the double-blind method.^4–9^

### Statistical Methods

RevMan 5.2, which was provided by the Cochrane Collaboration, was used for the meta-analysis. Because all endpoints were continuous variables, the data were expressed as weight mean difference (WMD) and 95% confidence intervals (95% CIs). To improve the analysis of the effect of ivabradine on angina pectoris, the patients were grouped according to follow-up time (<3 months group vs ≥3 months group) and the use of placebo in the control group (placebo group vs BB group). The 4 variables employed in meta-analysis were heart rate at rest, heart rate at peak, exercise duration, and time of onset of angina.

The Breslow–Day chi-square test (*P* < 0.1) and the I^2^ statistic were calculated to test the heterogeneity of the studies. I^2^ < 25% was considered to indicate low heterogeneity; 25% < I^2^ < 50% was considered to indicate moderate heterogeneity; and when *P* > 0.1 and I^2^ > 50%, the heterogeneity was considered to be high.^11^ When I^2^ < 50%, the fixed effects Mantel–Haenzel model was used to analyze the data. When I^2^ > 50%, the random-effects model of DerSimonian and Laird was used. A funnel plot was used to evaluate publication bias.^12^

### Ethical Statement

As this meta-analysis was based on previously published studies, ethical approval was not necessary.

## RESULTS

### Study Sample Selection

As shown in the flow diagram (Figure 1), 791 ivabradine-related articles were identified. After removing duplicates, 668 papers remained. Of these, 202 related to the treatment of stable angina pectoris with ivabradine. After removing the articles without a record of the 5 endpoints, 54 papers remained. After further screening, another 47 articles were excluded. Thus, 7 articles were selected for meta-analysis.^3–9^ These 7 studies had 3747 patients in total. Of these, 2100 were treated with ivabradine and 1647 were treated with control regimens. The patient characteristics are shown in Table 1.

### Risk of Bias in the Selected Studies

**FIGURE 2:**
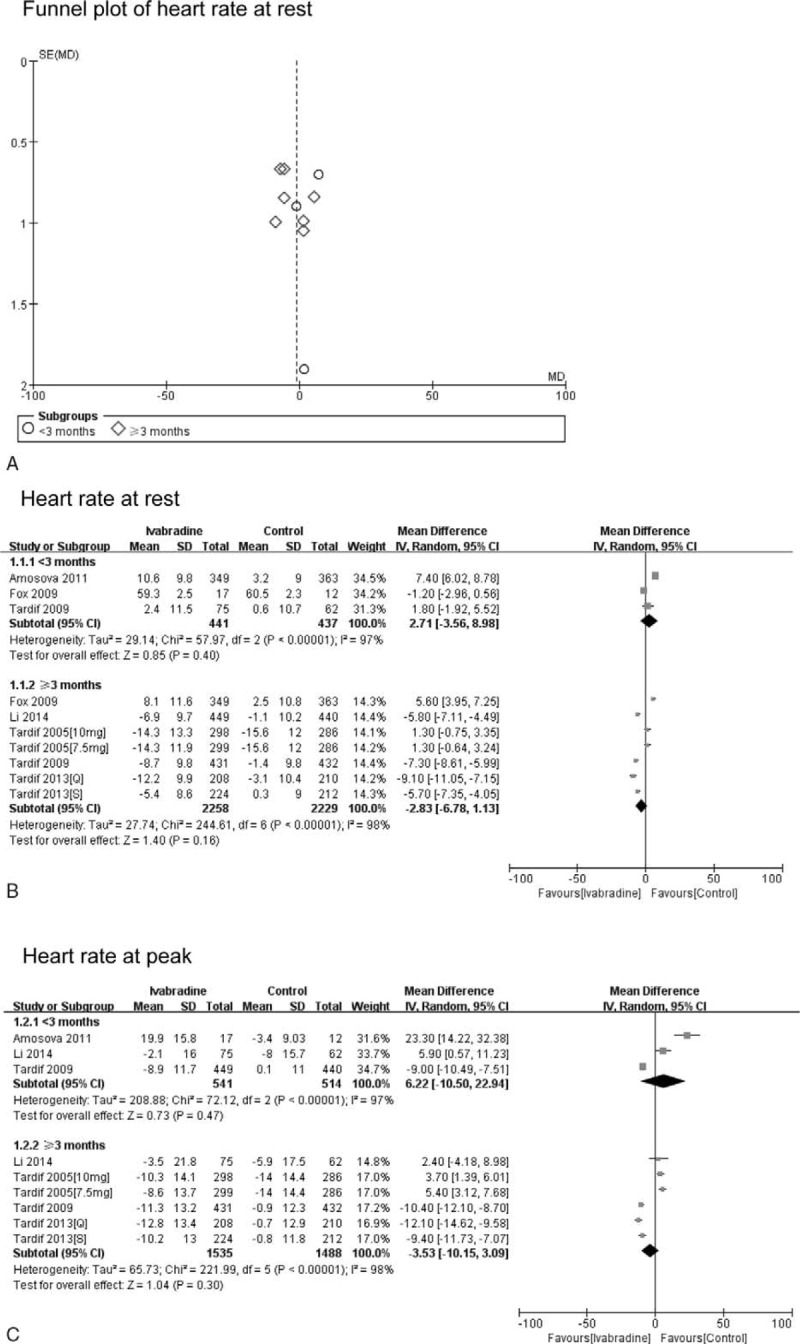
The patients were classified according to whether they were treated with ivabradine for less than or at least 3 months. The effect of different durations of treatment on heart rate at rest and at peak was assessed. A, Funnel plot of heart rate at rest. B and C, Forest plot of studies evaluating heart rate at rest (B) and at peak (C) (7.5 mg: treatment with ivabradine 7.5 mg bid; 10 mg: treatment with ivabradine 10 mg bid; Q: resting heart rate >65 bpm; S: resting heart rate ≤65 bpm).

In all, 7 RCTs were included, of which 1 was performed by the single-blind method and 6 by the double-blind method (Table 2). Since the funnel plot was approximately symmetrical, the 7 studies had little bias (Figure 2A).

**TABLE 2:**
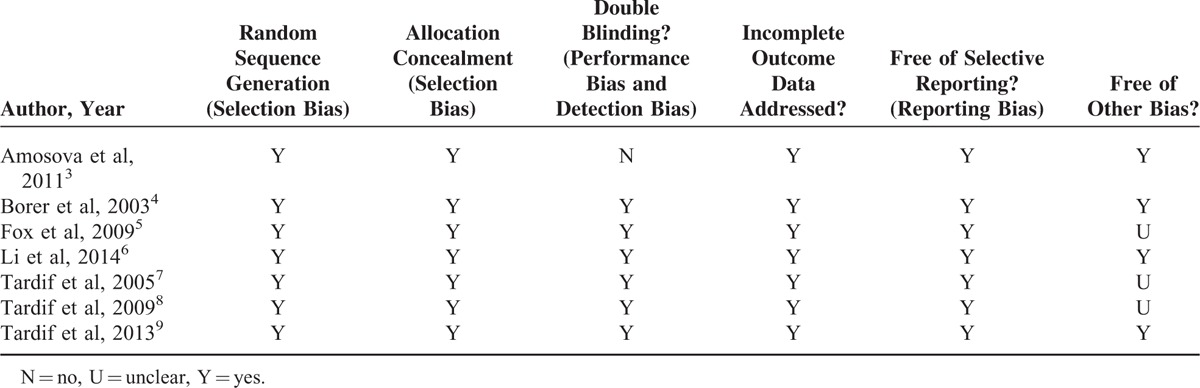
Methodological Quality Assessment of the Included Articles

### Effectiveness Indicators

#### Relationship Between Different Durations of Ivabradine Treatment and Effectiveness in Stable Angina

The 3747 patients were classified according to whether the ivabradine treatment had been for less than or at least 3 months, and the effect of different treatment durations on the following outcome variables was assessed.

*Heart rate at rest:* Three RCTs reported heart rate at rest after less than 3 months of treatment. Since there was significant heterogeneity (*P* < 0.01, I^2^ = 97%), the random-effects model of DerSimonian and Laird was used for data analysis. In patients treated for less than 3 months, the ivabradine and control groups did not differ significantly in terms of heart rate at rest (WMD = 2.71, 95% CI −3.56 to 8.98, *P* = 0.40). Five RCTs reported heart rate at rest after at least 3 months of treatment, of which 2 involved 2 experimental groups.^7,9^ In the patients treated for at least 3 months, the ivabradine and control groups did not differ significantly in terms of heart rate at rest (WMD = −2.83, 95% CI −6.78 to 1.13, *P* = 0.16) (Figure 2B).

*Heart rate at peak:* Three RCTs reported heart rate at peak after less than 3 months of treatment. In patients treated for less than 3 months, the ivabradine and control groups did not differ significantly in terms of heart rate at peak (WMD = 6.22, 95% CI −10.50 to 22.94, *P* = 0.47). Four RCTs reported heart rate at peak after at least 3 months of treatment, of which 2 involved 2 experimental groups.^7,9^ In the patients treated for at least 3 months, the ivabradine and control groups did not differ significantly in terms of heart rate at peak (WMD = −3.53, 95% CI −10.15 to 3.09, *P* = 0.30) (Figure 2C).

**FIGURE 3:**
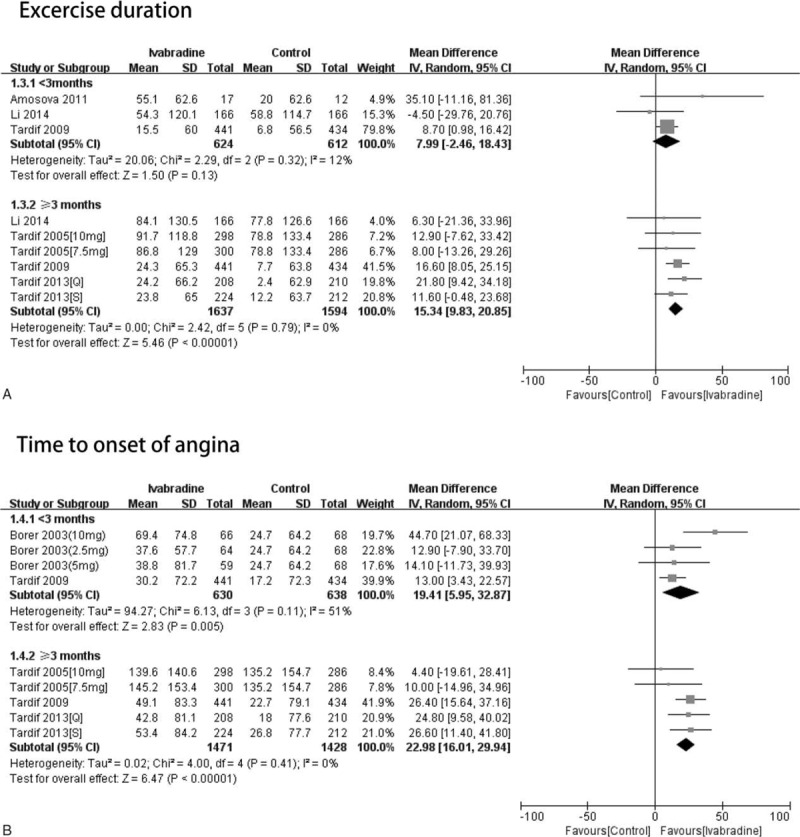
The patients were classified according to whether they were treated with ivabradine for less than or at least 3 months. The effect of different durations of treatment on exercise duration, and time to onset of angina was assessed. A and B, Forest plot of studies evaluating exercise duration (A) and time to onset of angina (B) (2.5 mg: treatment with ivabradine 2.5 mg bid; 5 mg: treatment with ivabradine 5 mg bid; 7.5 mg: treatment with ivabradine 7.5 mg bid; 10 mg: treatment with ivabradine 10 mg bid; Q: resting heart rate >65 bpm; S: resting heart rate ≤65 bpm).

*Exercise duration:* Three RCTs reported exercise duration after less than 3 months of treatment. In patients treated for less than 3 months, the ivabradine and control groups did not differ significantly in terms of exercise duration (WMD = 7.99, 95% CI −2.46 to 18.43, *P* = 0.13). Four RCTs reported exercise duration after at least 3 months of treatment, of which 2 involved 2 experimental groups.^7,9^ In the patients treated for at least 3 months, the ivabradine and control groups differed significantly in terms of exercise duration (WMD = 15.34, 95% CI 9.83–20.85, *P* < 0.01) (Figure 3A).

*Time to onset of angina*: Two RCTs reported time to onset of angina after less than 3 months of treatment, one of which involved 3 experimental groups.^4^ In patients treated for less than 3 months, the ivabradine and control groups differed significantly in terms of time to onset of angina (WMD = 19.41, 95% CI 5.35–32.87, *P* < 0.01). Three RCTs reported time to onset of angina after at least 3 months of treatment, of which 2 involved 2 experimental groups.^7,9^ In the patients treated for at least 3 months, the ivabradine and control groups differed significantly in terms of time to onset of angina (WMD = 22.98, 95% CI 16.01–29.94, *P* < 0.01) (Figure 3B).

#### Effectiveness of Ivabradine in Stable Angina When the Ivabradine Group Was Compared With Either Placebo or BB-receiving Controls

The 3747 patients were classified according to whether placebo or BB was used in the control group. The effect of the different controls on the following outcome variables was assessed.

**FIGURE 4:**
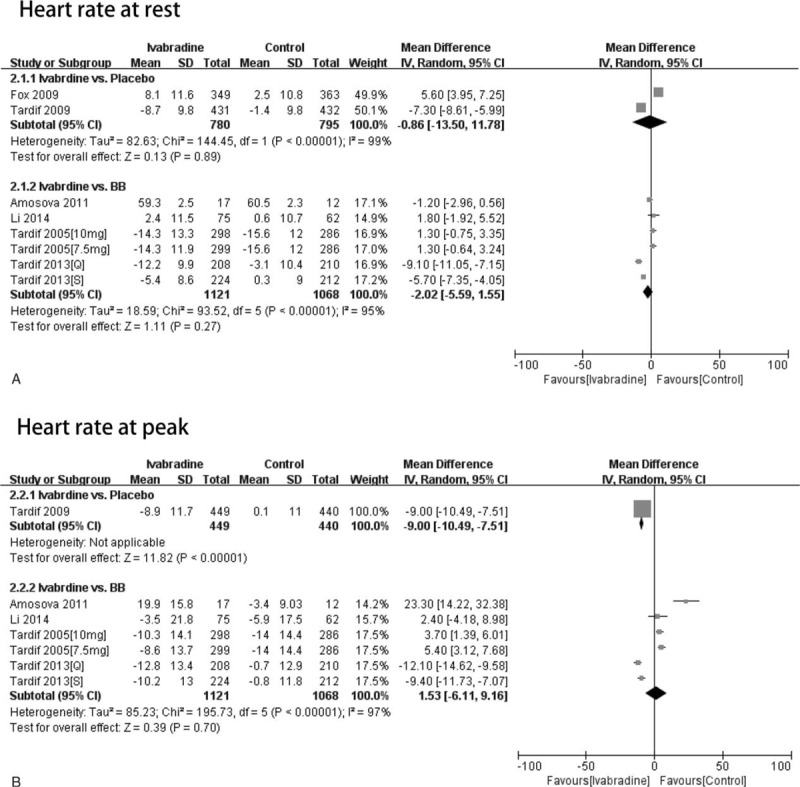
The patients were classified according to whether placebo or beta-blocker was used in the control group. The effect of the different controls on the heart rate at rest or at peak was assessed. A and B, Forest plot of studies evaluating heart rate at rest (A) and at peak (B) (7.5 mg: treatment with ivabradine 7.5 mg bid; 10 mg: treatment with ivabradine 10 mg bid; Q: resting heart rate >65 bpm; S: resting heart rate ≤65 bpm).

*Heart rate at rest:* Two RCTs reporting heart rate at rest used placebo-receiving controls. Because significant heterogeneity occurred (*P* < 0.01, I^2^ = 97%), the random-effects model of DerSimonian and Laird was used for data analysis. The ivabradine-treated and placebo-receiving patients did not differ significantly in terms of heart rate at rest (WMD = −0.86, 95% CI −13.50 to 11.78, *P* = 0.89). Two RCTs reporting heart rate at rest used BB-treated controls, of which 2 involved 2 experimental groups.^7,9^ The ivabradine and BB-treated groups did not differ significantly in terms of heart rate at rest (WMD = −2.02, 95% CI −5.59 to 1.55, *P* = 0.27) (Figure 4A).

*Heart rate at peak:* Only 1 RCT reporting heart rate at peak used placebo-receiving controls. The ivabradine-treated and placebo-receiving patients differed significantly in terms of heart rate at peak (WMD = −9.00, 95% CI −10.49 to −7.51, *P* < 0.01). Four RCTs reporting heart rate at peak used BB-treated controls, of which 2 involved 2 experimental groups.^7,9^ The ivabradine and BB-treated groups did not differ significantly in terms of heart rate at peak (WMD = 1.53, 95% CI −6.61 to 9.16, *P* = 0.70) (Figure 4B).

**FIGURE 5:**
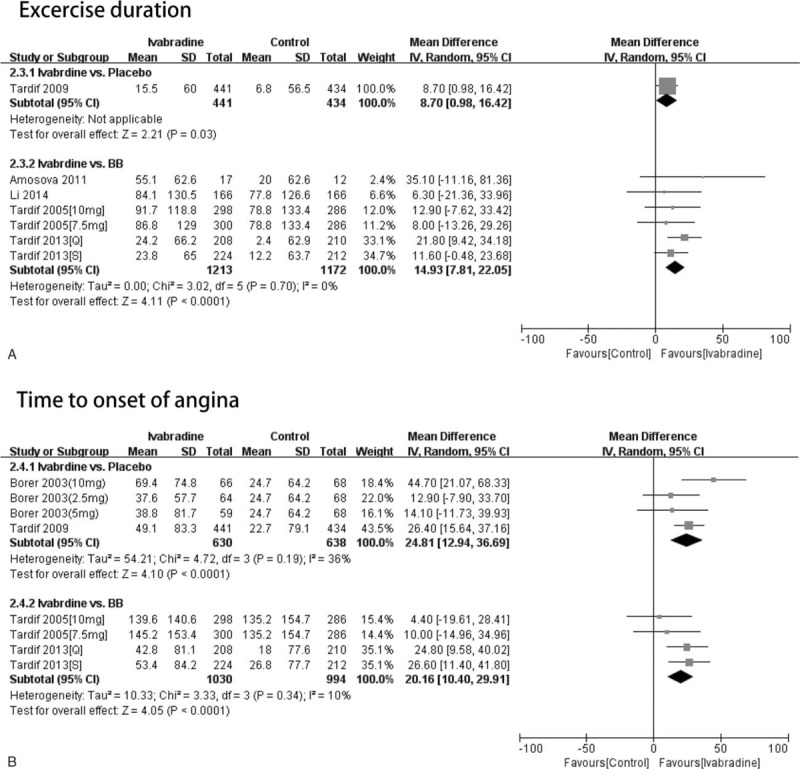
The patients were classified according to whether placebo or beta-blocker was used in the control group. The effect of the different controls on the exercise duration, time to onset of angina, and time to limiting angina was assessed. (A–B) Forest plot of studies evaluating exercise duration (A), and time to onset of angina (B). (2.5 mg: treatment with ivabradine 2.5 mg bid; 5 mg: treatment with ivabradine 5 mg bid; 7.5 mg: treatment with ivabradine 7.5 mg bid; 10 mg: treatment with ivabradine 10 mg bid; Q: resting heart rate >65 bpm; S: resting heart rate ≤65 bpm).

*Exercise duration:* Only 1 RCT reporting exercise duration used placebo-receiving controls.^8^ The ivabradine-treated and placebo-receiving patients differed significantly in terms of exercise duration (WMD = 8.70, 95% CI 0.98–16.42, *P* = 0.03). Four RCTs reporting exercise duration used BB-treated controls, of which 2 involved 2 experimental groups.^7,9^ The ivabradine and BB-treated groups differed significantly in terms of exercise duration (WMD = 14.93, 95% CI 7.81–22.05, *P* < 0.01) (Figure 5A).

*Time to onset of angina:* Two RCTs reporting time to onset of angina used placebo-receiving controls; 1 involved 3 experimental groups.^4^ The ivabradine-treated and placebo-receiving patients differed significantly in terms of time to onset of angina (WMD = 24.81, 95% CI 12.94–36.69, *P* < 0.01). Two RCTs reporting time to onset of angina used BB-receiving controls, of which 2 involved 2 experimental groups.^7,9^ The ivabradine and BB-treated groups differed significantly in terms of time to onset of angina (WMD = 20.16, 95% CI 10.40–29.91, *P* < 0.01) (Figure 5B).

## DISCUSSION

Angina is a clinical syndrome that is caused by acute and temporary myocardial hypoxia. Stable angina pectoris is defined as chest pain that develops when the heart rate rises, for example, during intense sports or exciting emotions. In such situations, the blood supply cannot meet the myocardial metabolism needs, thus leading to angina. The incidence of stable angina pectoris is increasing every year due to improving living standards and the aging of the population. Since angina seriously impairs patient quality of life, its symptoms should be treated. An important way to treat stable angina pectoris is to control the heart rate. Since angina pectoris is also an early manifestation of CHD, which can lead to myocardial infarction and heart failure, heart rate-reducing treatments may also reduce the incidence of ischemic heart disease, which is a common cause of death.^13,14,15^ This notion is supported by a recent meta-analysis showing an increased heart rate directly increases all-cause and cardiovascular disease mortality.^16^

There are a number of classical antiangina drugs such as BB that decrease heart rate,^17^ improve angina, and result in a better prognosis. However, BB also produces adverse reactions such as reducing atrioventricular conduction and inducing asthma. Ivabradine is an alternative to BB that has recently received widespread attention because of its ability to specifically decrease the heart rate. Several large clinical trials have confirmed that it effectively treats heart failure.^18,19^ As a result, the 2012 guidelines on the diagnosis and treatment of heart failure by the European Society of Cardiology recommend ivabradine as a first-line treatment.^20^

A pilot study^21^ showed that ivabradine may be used safely to decrease the heart rate in acute ST-segment elevation myocardial infarction. However, ivabradine does not seem to be as potently curative in heart failure: when Fox et al^22^ summarized the data from the BEAUTIFUL and SHIFT trials on the curative effect of ivabradine in 11,897 patients with left ventricle dysfunction and heart rate ≥70 bpm, they found that the ivabradine and placebo groups did not differ significantly in terms of cardiovascular mortality and total mortality, although ivabradine did reduce the relative risk for the composite of cardiovascular mortality, heart failure hospitalizations, and myocardial infarction hospitalizations. Since ivabradine seems to have a significant curative effect on angina and myocardial infarction, its relatively poor curative effect in heart failure means that it remains unclear whether using ivabradine to treat angina pectoris can actually prevent future heart failure. However, several clinical trials^3,23–25^ show that ivabradine combined with other drugs has a significant curative effect in terms of treating angina pectoris and future heart failure. Thus, studies that have long follow-up durations are needed to identify the ivabradine-including treatment regimens that both prevent angina pectoris and reduce the subsequent development of heart failure.

Twenty-three RCTs were included in the meta-analysis by Cucherat and Borer,^26^ of which 2 on ivabradine indicated that ivabradine can slow heart rate; however, the other 2 important indicators (exercise duration and time to onset of angina) were not analyzed. In the meta-analysis by Belsey et al,^27^ only 1 RCT^8^ reported that ivabradine can improve the result of exercise tolerance test. To further clarify how effective ivabradine is in treating angina pectoris, only relevant RCTs were collected for the meta-analysis described in the present study.

The present meta-analysis showed that the ivabradine-treated and control groups did not differ in terms of heart rate at rest or at peak regardless of whether the treatment was for less than 3 or at least 3 months. The 2 groups also did not differ in terms of exercise duration when they were treated for less than 3 months. However, when treatment was for at least 3 months, the ivabradine-treated group had better exercise duration than the control group. Thus, to improve exercise duration, patients with stable angina should keep taking ivabradine for more than 3 months. The ivabradine-treated group had longer times to onset of angina than the control group regardless of whether the treatment duration was short (less than 3 months) or long (at least 3 months).

The present meta-analysis also showed that the ivabradine-treated patients had similar heart rates at rest regardless of whether they were compared with placebo or BB-receiving control groups. However, in terms of heart rate at peak, the ivabradine-treated group had better values than the placebo-receiving group, but not the BB-treated controls. The ivabradine-treated patients had better exercise duration and time to onset of angina than both the placebo and BB-receiving controls.

This study had a number of advantageous features. Firstly, the sample size was large (N = 3748). Secondly, a meta-analysis only on the efficacy of ivabradine in stable angina has not yet been published. Thirdly, the study results indicate that further focus should be placed on the effect of ivabradine treatment on angina duration. Fourthly, this meta-analysis assessed both the effect of different treatment durations and the effect of different comparator control groups on the efficacy of ivabradine for treating stable angina pectoris.

This study also had several limitations. Firstly, it was based on 7 articles only. Additional well-designed studies are needed to validate the findings of this meta-analysis. Secondly, only 4 indicators of curative effect were analyzed in this study. As a result, the effect of ivabradine on stable angina pectoris was not fully determined. Thirdly, the 7 studies differed in the dose of ivabradine. Fourthly, the studies differed in terms of treatment strategies. For example, in 1 study, ivabradine was only used after treatment with other drugs.^3^ These considerations may affect the accuracy of the present meta-analysis.

A number of issues remain to be addressed by further research. Firstly, although ivabradine therapy effectively treats angina pectoris, can it prevent future heart failure? Secondly, does long-term use of ivabradine have a better prognosis than short-term use? Thirdly, would combination therapies employing ivabradine together with other drugs be more effective in treating angina pectoris than therapy based on ivabradine alone?.

## CONCLUSIONS

Compared with BB and placebo, ivabradine improved the exercise duration and time to onset of angina in patients with stable angina. However, its ability to improve exercise duration only became significant after at least 3 months of treatment.
